# Overseas Chinese Returnees’ Swindler Syndrome and Their Entrepreneurial Education Under Psychological Resilience

**DOI:** 10.3389/fpsyg.2021.747687

**Published:** 2022-01-07

**Authors:** Can Xiao, Xiaoya Wang

**Affiliations:** College of Education, Washington State University, Pullman, WA, United States

**Keywords:** psychological resilience, overseas Chinese returnees, entrepreneurship education, cheating syndrome, Chinese returnees’ entrepreneurship

## Abstract

The study aims to explore the entrepreneurship education of overseas Chinese returnees with the swindler syndrome through psychological resilience. First, a questionnaire survey is conducted to analyze the current situations of entrepreneurship education of overseas Chinses returnees and college students, and it is found that the entrepreneurship education received by overseas Chinese returnees is more advanced and perfect than that by domestic students, which makes overseas Chinese returnees have the ability to solve the problems in the process of entrepreneurship, realizing their entrepreneurial dream. However, the emergence of swindler syndrome changes the self-awareness and psychology of these returnees, which is improved through appropriate entrepreneurship education under resilience analysis. The results show that entrepreneurial resilience and entrepreneurial optimism covered by psychological resilience have a significant positive impact on entrepreneurial intention, indicating that entrepreneurial resilience and entrepreneurial optimism can enhance individual’s entrepreneurial intention. The scores of the subjects with the experience of studying abroad are higher than those without such experience, indicating that overseas Chinese returnees have stronger resilience and more optimistic attitudes in the face of difficulties and setbacks, which provides a new perspective for in-depth analysis of Chinese returnees’ entrepreneurship education and promotes the development of entrepreneurship education in colleges and universities in China.

## Introduction

With the development of economic globalization and the advent of the information age, the entrepreneurial activities of college students are increasing in recent years and gradually become an important driving force for the change of the global economic and industrial pattern. At present, China has entered into a period of economic transformation, and entrepreneurial activities bring more jobs or high-yield industries. Therefore, under the support of China’s economic development transformation period and the policy of overseas Chinese returnees’ entrepreneurship, the swindler syndrome and entrepreneurship education of overseas Chinese returnees in entrepreneurship (Van and Weber, 2021) are analyzed and studied. With the development of China’s economy, more and more overseas Chinese returnees tend to return to their homeland and start a business. Their experience of studying and living abroad provides a lot of help in the process of entrepreneurship, and also promotes the development of China’s social economy. Also, the survey of entrepreneurship education during their study abroad shows that entrepreneurship education provides theoretical knowledge for domestic universities (Lambert and Rennie, 2021).

The psychological term of the swindler syndrome is a phenomenon or negative tendency of self-ability, which makes entrepreneurs feel that their success in starting a business has nothing to do with their ability. In 1978, clinical psychologists Dr. Pauline Cransey and Susanna Ems find that women who have achieved success often attribute their success to luck, opportunity, or confidence given by others. Therefore, they feel that their success is deceptive and they do not deserve success. They defined this phenomenon as “Swindler Syndrome,” which is not actually equivalent to being a liar who often deceives others. Facts prove that the group with swindler syndrome is really capable, knowledgeable, or skillful, and they are good at doing their own things. They do not believe in their success, that is, they cannot internalize their achievements even if all the evidence proves that they are excellent. Anyone may suffer from “Swindler Syndrome,” which usually has in groups with outstanding achievements in various fields. According to statistics, about 70% of people have different degrees of “Swindler Syndrome” at some stage of their lives. As a psychological term, resilience is a scientific and intuitive method to reflect the psychological changes of students when they start a business (Moradi, 2021). Moreover, resilience is a special psychological trait between subjects and objects, and it changes greatly with the change of the environment and cultural and educational backgrounds, which enables researchers to feel the differences between overseas Chinese returnees and domestic college students in the entrepreneurial process (Seaborn et al., 2021). Through the review of the relevant literature, the problems that need to be paid attention to the entrepreneurship education of college students in China are explored, and the characteristics of students’ swindler syndrome reveal the factors affecting students’ entrepreneurship and provide help for the development of entrepreneurship education in China.

This study is divided into four parts, the first part is the introduction, in which the research background, significance, and purpose are briefly introduced; the second part mainly analyzes the theories and methods involved, including the theories of “Swindler Syndrome” and psychological resilience. The third part discusses the influence of psychological resilience on entrepreneurial resilience through the results of the questionnaire survey. The fourth part summarizes the content of this article. Based on psychological resilience, overseas Chinese returnees’ entrepreneurial swindler syndrome and entrepreneurship education are analyzed. The role of psychological resilience in entrepreneurship education is studied, and the causes of the swindler syndrome are discussed. Through the analysis and research of their psychological resilience, the relationship of overseas Chinese returnees’ experience, swindler syndrome, and entrepreneurship education is analyzed, and the role of international student’s experience in the process of entrepreneurship is discussed, which provides a reference for entrepreneurship education in China. The innovation of the study lies in the analysis of overseas Chinese returnees’ entrepreneurial experience, the study of the swindler syndrome, and entrepreneurship education based on psychological resilience, which provides a new perspective for entrepreneurship education and the students’ swindler syndrome in colleges and universities, promoting the development of entrepreneurship education in China.

## Relevant Theories and Methods

### Swindler Syndrome

The swindler syndrome is a new concept proposed in the 1980s. It refers to a feeling that individuals do not believe that their success comes from their great effort or ability and that they have no talent and are not qualified for anything they want, or they are afraid to be considered as “liars” in some cases. It is a psychological term and considered as the impostor phenomenon. Previous studies reveal that many females with great achievements often feel that she herself is a liar. They attribute their success to good luck, or they feel that they cheat others in some way, which makes them feel their success is fraudulent and untrustworthy. This is different from the behavior of deceiving others intentionally. Also, it does not mean that people who pretend to be successful before they succeed. In addition, it is not equal to self-doubt. Actually, the swindler syndrome group is successful and they are good at what they do, which has been proved by external objective evidence. The problem is that they simply do not believe their efforts and abilities. More precisely, they cannot face up to their achievements. Instead, all their achievements are attributed to chance coincidence or other people’s kindheartedness, not to their hard work and long-term dedication (Sotiropoulos, 2021; Campbell et al., 2021). There is a case in which MacArthur Wheeler was found shortly after committing robbery without any disguise, but he thought he was wise to use lemon juice. This case attracts wide attention from the psychological research team. In different situations, people’s attitudes toward things are different. They always make certain behaviors by consciousness at the first time of seeing things, rather than through rational thinking. Some entrepreneurs also attribute their entrepreneurial success to coincidence, their hard work, and their effort. They think that anyone can do it even if all the evidence shows that they are excellent at doing the task. Based on psychological resilience, the swindler syndrome of the returned entrepreneurs is analyzed, and some psychological states of the entrepreneurs in the process of entrepreneurship are analyzed (Regan et al., 2020).

### Overview of Overseas Chinese Returnees’ Studying Experience

An international student refers to a student who goes abroad and receives education abroad. Education abroad can be divided into short-term and long-term studies according to the study time. The time spent in foreign countries can be several weeks, months, or years. Those with the experience of studying abroad are called overseas Chinese returnees. They experience different cultures in different countries and receive different styles and methods of education, which is a special kind of an individual’s experience in education and has an impact on individuals’ personality and behavior (Sutherland et al., 2021).

**Figure 1:**
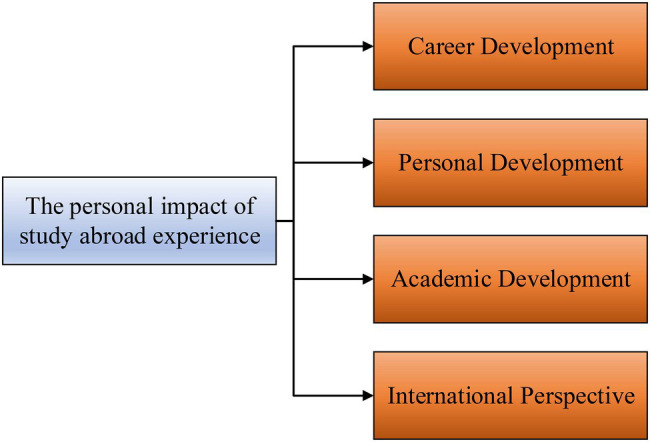
Impact of study abroad experience on overseas Chinese returnees.

Overseas Chinese returnees usually experience different school environments, teaching modes, and extracurricular activities, as well as cross-cultural adaptation and different social situations. Among them, cross-cultural adaptation is reflected in all aspects of overseas Chinese returnees’ life, such as the adaptation to local culture, customs, and the relationship with local people, which may change the life of overseas Chinese returnees. Cross-cultural adaptation has a very close relationship with the study and life of overseas Chinese returnees, and it is the focus of research in the field of education and psychology. The key situations are analyzed in the experience of studying abroad, the current situation of entrepreneurship education and swindler syndrome of overseas Chinese returnees are discussed, and their experiences are explored (Rabeeah et al., 2021; Lim and Chen, 2021). The impact of the experience of studying abroad on overseas Chinese returnees is mainly reflected in the following aspects, as shown in Figure 1.

Based on the analysis of overseas Chinese returnees’ study experience and entrepreneurship education, the problems encountered by overseas Chinese returnees in the process of entrepreneurship are discussed, and the influence of overseas Chinese returnees’ characteristics, their innovation, and advantages in entrepreneurship are compared with those of the students without overseas experience. Psychological resilience is introduced to analyze the relationship between overseas Chinese returnees’ experience and entrepreneurship, which has great significance in the construction of relevant theoretical models.

### Theory of Psychological Resilience and Its Dimension Division

**Figure 2:**
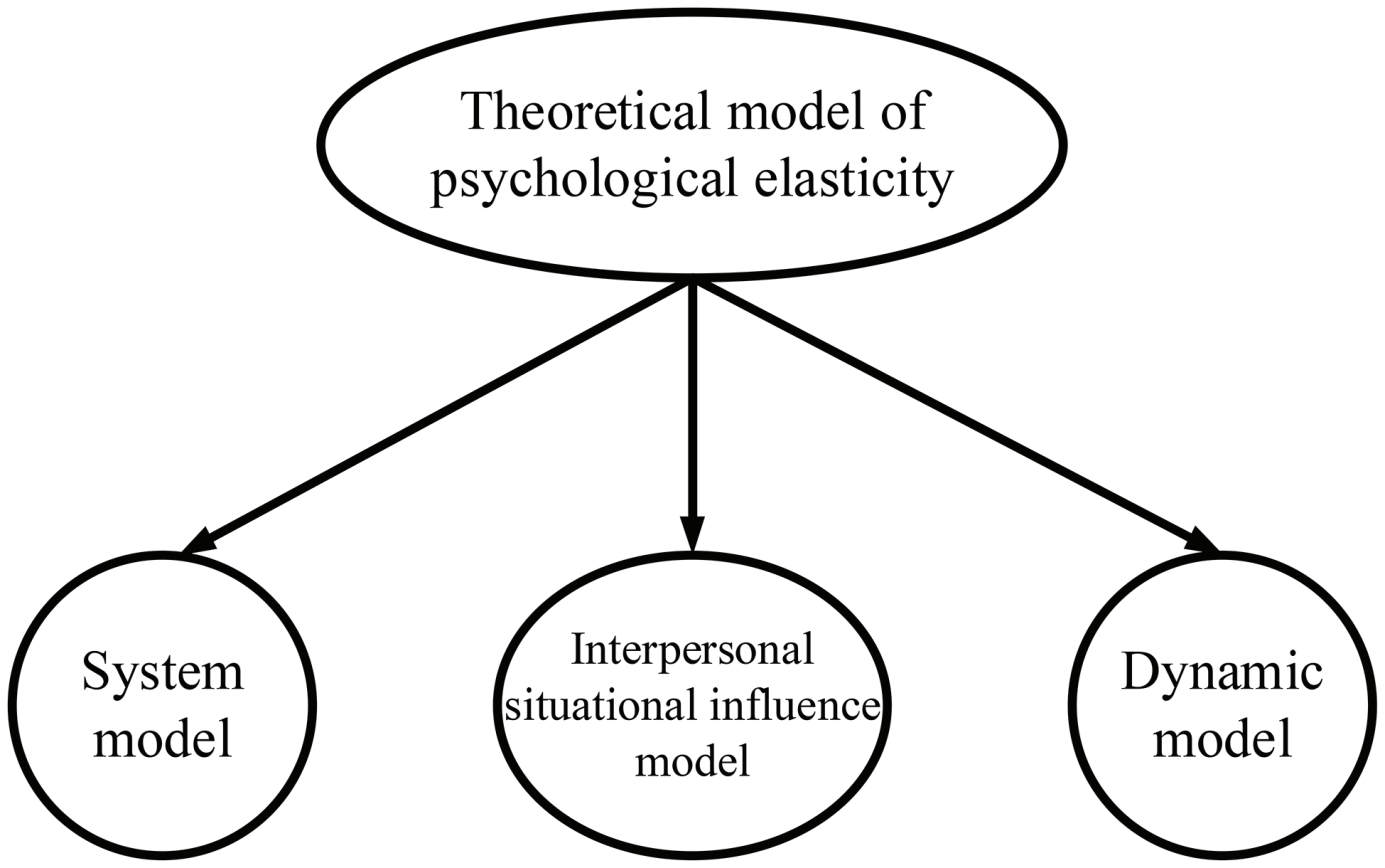
Theoretical model of resilience.

Resilience is produced by various protective and risk factors through interaction. When an individual has relatively strong psychological resilience, they can be stable in communication, emotional control, and solving problems (Riedel et al., 2020). In terms of the role of resilience, the researchers construct a theoretical model of resilience that effectively matched the types of research subjects. Figure 2 is the theoretical model of resilience.

#### System Model

Taking children as the research subjects, some research teams construct the children’s resilience model (Kotera and Ting, 2021). In the study, the external factors are divided into two kinds, namely, the external and internal factors of the family. Schools and teachers belong to the external factors of the family, while relatives and family conditions are the internal factors of the family. Individual internal factors can also be divided into two kinds, namely, psychological and biological factors. Individual cognitive ability, problem-solving ability, and personality are psychological factors. Physical health and gender are objective factors (Yang et al., 2021). The above factors are interrelated and can be in a state of dynamic equilibrium. When an individual lack one, individuals with relatively strong resilience can rely on other factors to supplement in a very short time.

#### Interpersonal Model

According to the interpersonal model, it is found that psychological activities like individual perception are affected and changed by different situations, which enables individuals to show differences in behavior, and then feedback to the scene after the implementation of the behavior. The actual impact mechanism can be divided into four aspects, namely, situation, self, action, and result. Situations refer to social support, parental involvement, poverty, and other factors, and they are the influences of the surrounding environments on psychological characteristics (Cagla and Nurcan, 2021). Self refers to individual temperament, individual perception and relationship, and individual imitation ability. Action refers to the performance of situations and actions, and it is a specific idea of whether to take measures. The result is the effect of the combined action of situations, selves, and actions. The result may be negative or positive, and the manifestations may be depression, anti-social behavior, or prosocial behavior.

**Figure 3:**
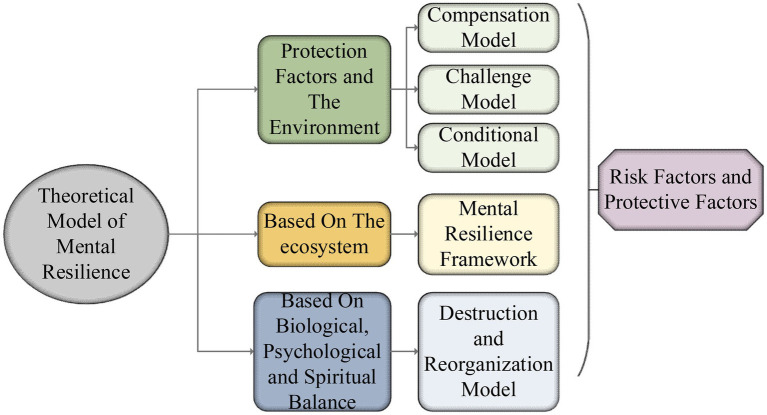
The dimension division of psychological resilience.

In addition, the difference of resilience in individuals exists not only in the individual’s congenital differences but also in the influence of acquired education, environment, and practice. Therefore, resilience should be the result of the interaction of congenital and acquired. Moreover, there is a positive correlation between resilience and individual adaptability, that is, the greater the resilience is, the stronger the individual’s ability to regulate the external environment is (Lau et al., 2021). There are two main dimensions of resilience, as shown in Figure 3.

#### Dynamic Model

The psychodynamic model is first introduced into the study of practical problems by psychologists and related scholars in California, the United States in 2003. The research subject of this model is teenagers. The study proposes the sense of belonging, respect, and other belonging to the psychological needs of teenagers in the growth period, so the resilience of teenagers in the process of continuous growth always changes dynamically. Family and social environments are the key influencing factors of a teenager’s resilience, and they can help to form individual traits, such as self-efficacy and self-awareness.

The above three theoretical models have different perspectives on the mechanism of resilience, but they also have similarities, that is, resilience is caused by the interaction of various protective factors. In this regard, the system model pays more attention to the impact of external and internal factors. Therefore, the internal factors of the theory are drawn on, the impact of this factor on entrepreneurial intention is considered, and the factor of entrepreneurial resilience is added to do the research. The interpersonal model focuses on the role of social interpersonal communication and regards it as a key factor affecting the self-cognition of individual members. In addition, concerning the external influencing factors of this theory, the dimension of entrepreneurial optimism is added to the design of the variable of psychological resilience. The dynamic model puts forward the key role of external factors on resilience. This model provides an effective reference for subsequent research (Zhou et al., 2021).

### Theoretical Model of Entrepreneurship Education

With the development of higher education, the education for college students encounters unprecedented difficulties, which affects economic development. Therefore, entrepreneurship education turns to promoting the education of college students since it is not only conducive to economic development but also contributes to the smooth implementation of higher education. Entrepreneurship education is essential to cultivate students’ comprehensive quality of entrepreneurship, such as entrepreneurial awareness, entrepreneurial thinking, and entrepreneurial skills, enabling students to have a certain entrepreneurial ability. Entrepreneurship education is called the “third passport” of education by the United Nations Educational, Scientific and Cultural Organization, and is given the same important position as academic education and vocational education. Entrepreneurship education is not equal to the education of creating enterprises. In this regard, the British have a unique understanding. They believe that entrepreneurship is the process of taking responsibility, actively seeking and seizing opportunities, efficiently integrating and utilizing resources, wisely making decisions, creatively solving problems, and innovating and creating values in a chaotic, disorderly, changing, and uncertain environment. Entrepreneurship refers to goal attainment, as well as “creative destruction” sometimes. On this basis, entrepreneurship cannot be simply regarded as a purely profit-oriented business activity, but a way of thinking and behavior that permeates people’s lives. Entrepreneurial activities require college students to have the innovative spirit and qualities of autonomy, self-confidence, diligence, perseverance, courage, and integrity and require universities to cultivate students’ achievement motivation, pioneering spirits, and the ability to analyze and solve problems. The objective of entrepreneurship education is to cultivate students’ entrepreneurial skills and pioneering spirit, which can make them bravely respond to the challenges that globalization and knowledge economy bring, change their concepts of employment, and take entrepreneurship as an occupation. Entrepreneurship education should teach students not only knowledge and skills about entrepreneurship but also the thinking mode of entrepreneurs. Besides, entrepreneurship education is the basis of entrepreneurship, that is, through strict academic training and knowledge preparation, entrepreneurs should have a strategic vision, good communication and coordination ability, marketing ability and decision-making ability, and high emotional quotient (EQ). Entrepreneurship education is also called innovation and entrepreneurship education. It is a practical education aimed at cultivating talents with basic entrepreneurial qualities and pioneering personalities. In the process of entrepreneurship education, it is necessary to cultivate students’ entrepreneurial awareness, entrepreneurial spirit, entrepreneurial ability, and other entrepreneurial-related abilities and qualities. In other words, entrepreneurship education should comprehensively cultivate students from their awareness, abilities, the cognition of entrepreneurial environments, and the practice simulation (Paul et al., 2021).

Entrepreneurship education can also be called innovation and entrepreneurship education. It is a practical education aimed at cultivating talents with basic entrepreneurial qualities and pioneering personalities. In the process of entrepreneurship education, it is necessary to cultivate students’ entrepreneurial awareness, entrepreneurial spirit, entrepreneurial ability, and other entrepreneurial-related abilities and qualities. In other words, students’ entrepreneurship education should be carried out from the cultivation of their awareness, the improvement of their ability, the cognition of entrepreneurial environment, and practice (Paul et al., 2021).

**Figure 4:**
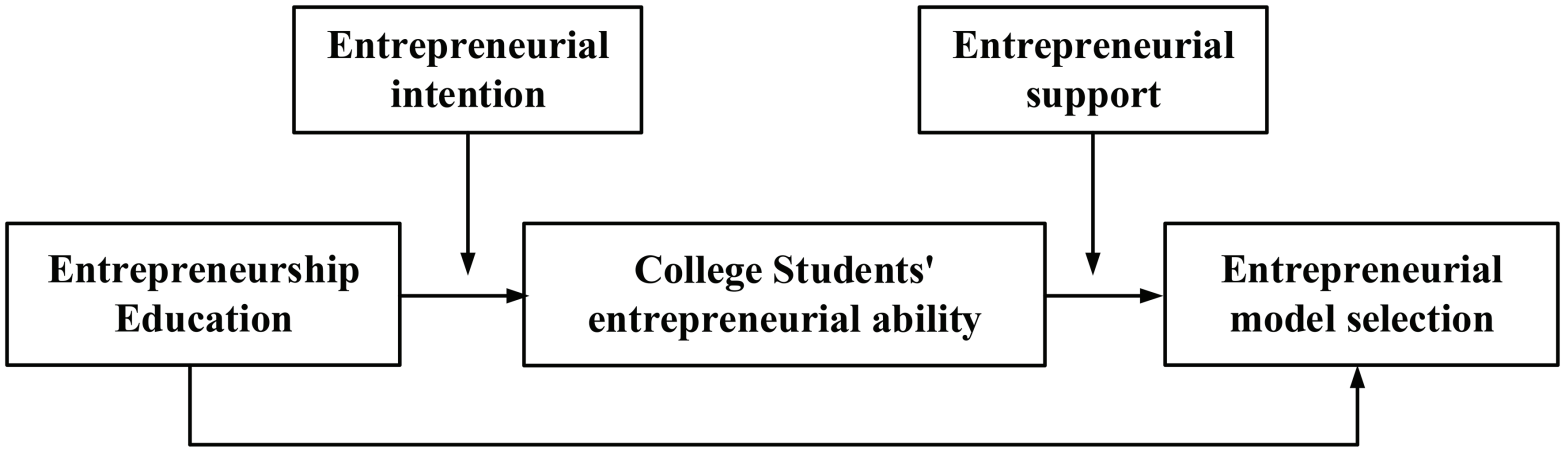
The research model.

Compared with entrepreneurship education abroad, entrepreneurship education in colleges and universities in China is relatively backward. This is shown by the differences in the performance of domestic college students and overseas Chinese returnees in the process of entrepreneurship (Liang et al., 2021). In the study, the entrepreneurship education of overseas Chinese returnees is discussed based on psychological resilience. The research model of entrepreneurship education is proposed according to the relevant literature. The model is shown in Figure 4.

Figure 4 shows that the entrepreneurial ability of college students is dependent on the entrepreneurial model and their entrepreneurship education received. The entrepreneurial support of all aspects affects the choice of entrepreneurial model of college students, and their entrepreneurial intention affects the implementation of entrepreneurship education. Therefore, the entrepreneurial intention of college students and all aspects of entrepreneurial support should be counted to discuss the entrepreneurial ability of college students.

## Research Methods

In addition to the literature research method, a questionnaires survey is conducted to analyze the entrepreneurial situation of overseas Chinese returnees. Through a pre-survey, the problems in the questionnaire are adjusted to ensure the accuracy of the survey results. After the recovery of the questionnaires, the invalid questionnaires are excluded. Through the test of the reliability and validity of the questionnaire, it is proved that the questionnaire is rational. Finally, the questionnaire is analyzed to study the relationship between the relevant variables (Şahin and Türk, 2021).

A total of 150 questionnaires are distributed to overseas Chinese returnees, and 142 valid questionnaires are collected, with an effective rate of 94.6%. Among them, 52.3% are science and engineering majors, 29.1% are economics and management majors, and 18.6% are other majors. Based on the authoritative scale in China and foreign countries, the Likert7 subscale is used to measure each variable (1 = inconsistent at all; 2 = quite inconsistent; 3 = inconsistent; 4 = uncertain; 5 = consistent; 6 = somewhat consistent; and 7 = completely consistent), and the steps are as follows: (1) entrepreneurship education is divided into two dimensions of curriculum education and practical education by dichotomy; (2) the research scale has six items, for example, “entrepreneurship education enriches my understanding of the role of entrepreneurs”; (3) four items are set to measure the variable of entrepreneurial intention by referring to the research scales of Fernández-Pérez and Virginia, for example, “I plan to start a business within 5 years after graduation.” The entrepreneurial model is divided into two types: independent entrepreneurship and collaborative entrepreneurship. According to the research hypotheses, four reverse items are set for the measurement, for example, “I need to cooperate with enterprises to implement entrepreneurial behavior.” Entrepreneurial support includes government support, business support, and family support. Six items are set to measure it by referring to the research scale of Malebana and JusticeM, for example, “family can provide some financial support for my entrepreneurship.” In addition, the scale also counts and controls the basic demographic information, such as gender, age, education, and patent. And SPSS 25.0 software is used for descriptive statistics of sample data, Mplus data processing software is used for the reliability and validity test, the model fitting analysis, and the test of mediating effect and moderating effect.

The most commonly used reliability test method, namely, Cronbach’s *α*, is used to measure and analyze the variables of the questionnaire. It is the average value of the reliability coefficient of all possible methods of the scale. The specific calculation equation is as follows.


$$\alpha =\frac{M}{M-1}\left(1-\frac{\sum s_{n}^{2}}{s^{2}}\right)$$

(1)

In equation (1), *M* is the number of items in the scale, $\sum s_{n}^{2}$ is the variance of the current sample in the scale, and *S*^2^ is the variance of the total sample in the scale.

In the validity analysis of the scale, factor analysis is carried out from several aspects of overseas Chinese returnees’ entrepreneurship education, and the research content is determined according to the results (Dong et al., 2021; Songül and Bahar, 2021). The followings are the content of the questionnaire.

### Entrepreneurship Education (EE)

EE1-EE7 are as follows: systematically take entrepreneurship courses and participate in entrepreneurship practice, Entrepreneurship education encourages me to create entrepreneurial ideas; Entrepreneurship education helps me form entrepreneurial preferences; Entrepreneurship curriculum provides necessary entrepreneurial knowledge; Entrepreneurship education enriches students’ cognition of the role of entrepreneurs; Entrepreneurship education deepens students’ understanding of the entrepreneurial process; and Entrepreneurial practice cultivates students’ entrepreneurial ability (Cara et al., 2019; IJntema et al., 2021).

### Entrepreneurial Capability (EC)

EC1-EC4 are as: be good at discovering products that have not been promoted in the field; be good at finding entrepreneurial opportunities; be good at using opportunities; and be good at using various resources.

### Entrepreneurial Model (EM)

EM1–EM7 are as: when I decide to start a business, I will choose to start a business independently; I believe I can start my own business; I have the resources and channels for self-employment; Compared with independent entrepreneurship, I prefer to choose collaborative entrepreneurship; I need to work with the company to achieve entrepreneurial goals; I need to cooperate with existing enterprises to implement entrepreneurial behavior; and Cooperating with successful enterprises can help me reduce entrepreneurial risks.

### Entrepreneurial Intention (EI)

EI1–EI4 are as: I plan to start a business within 5 years after graduation; My goal is to become an entrepreneur, and I am more inclined to start a business than employment; I have taken some actions to start a business; and Despite the risk of failure, I will continue to try to start a business until I succeed.

### Entrepreneurship Support (ES)

ES1–ES12 are as: the government will provide the necessary support if I decide to start a business; In the process of entrepreneurship, the government will issue a series of support policies; In the entrepreneurial process, I can get some financial support from the government; In the entrepreneurial process, the Government will provide information and infrastructure; the existing enterprises will provide help If I decide to start a business; In the entrepreneurial process, I will keep close contact with related enterprises; Enterprises can provide good financial and technical support for my entrepreneurship; In the entrepreneurial process, successful enterprises will share high-quality entrepreneurial experience and social networks; the family will give me support if I decide to start a business; family business background will provide experience support for my entrepreneurship; the family can provide some financial support for my entrepreneurship; and My family’s support has strengthened my faith in entrepreneurship (Li, 2021).

The questionnaire with the above items can reveal the role of resilience in overseas Chinese students’ entrepreneurship, show the current situation of entrepreneurship education received by overseas Chinese students, and analyze the entrepreneurship education of overseas Chinese returnees with the swindler syndrome, finding solutions and paths to the problems in entrepreneurship education.

The questionnaire with the above questions can show the role of psychological resilience in overseas Chinese returnees’ entrepreneurship, help to know about the current situation of the entrepreneurship education received by overseas Chinese returnees, and analyze the entrepreneurship education of foreign students and the situation of the swindler syndrome, figuring out the corresponding solutions and paths.

## The Results of the Questionnaire and the Analysis of Overseas Chinese Returnees’ Entrepreneurship

### Results of the Questionnaire

**Figure 5:**
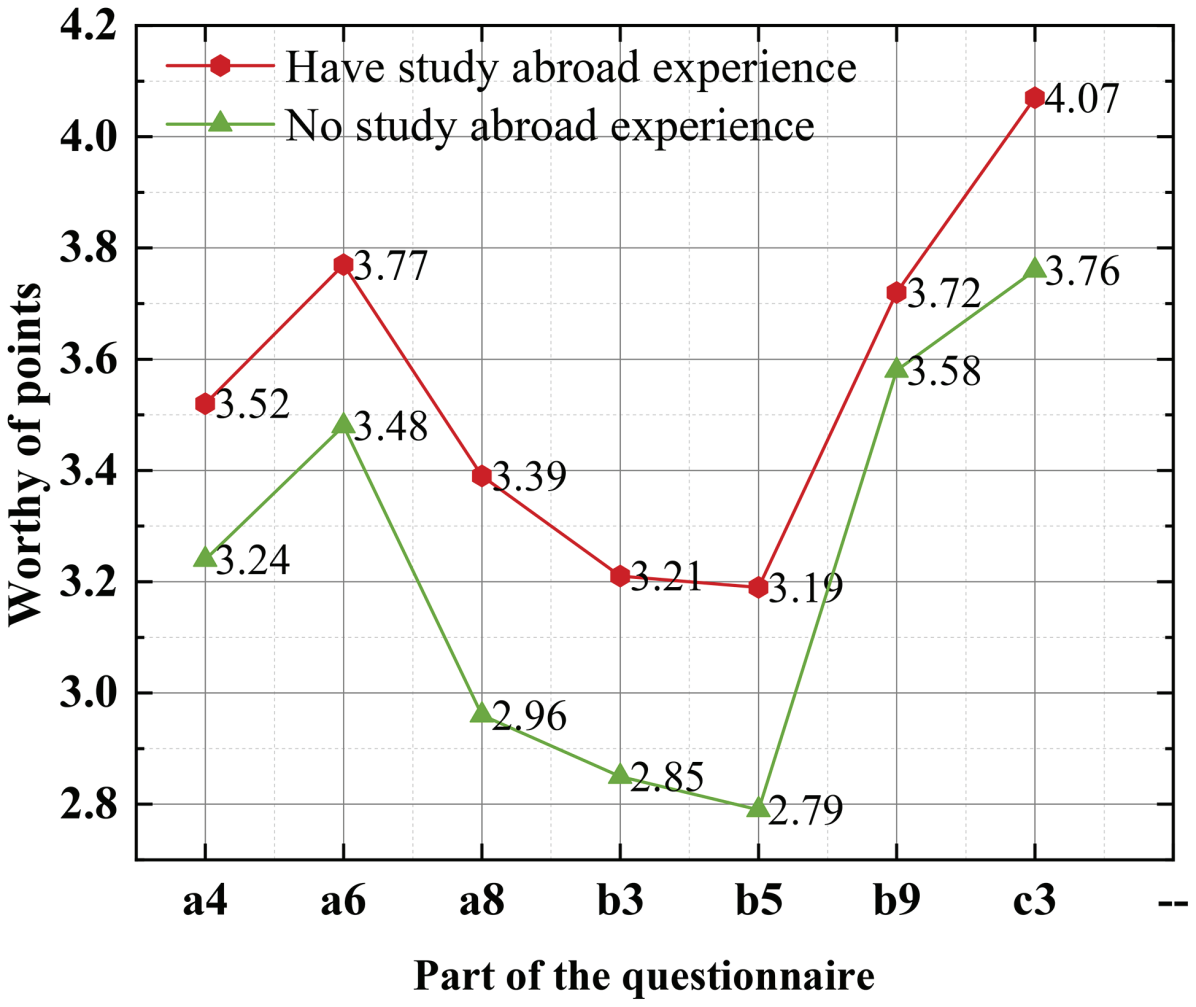
Mean standard deviation of the scores of the subjects.

The questionnaires are randomly distributed, and the scores of the subjects who have the experience of studying abroad or not are collated and calculated, respectively. The mean standard deviation of the scores of the subjects is shown in Figure 5.

Figure 5 shows the average score of the subjects with and without studying abroad experience. Specifically, the average score of the students with studying abroad experience is higher than that of the students without studying abroad experience on some questions in the questionnaire, which is also intuitively reflected in the figure.

**Figure 6:**
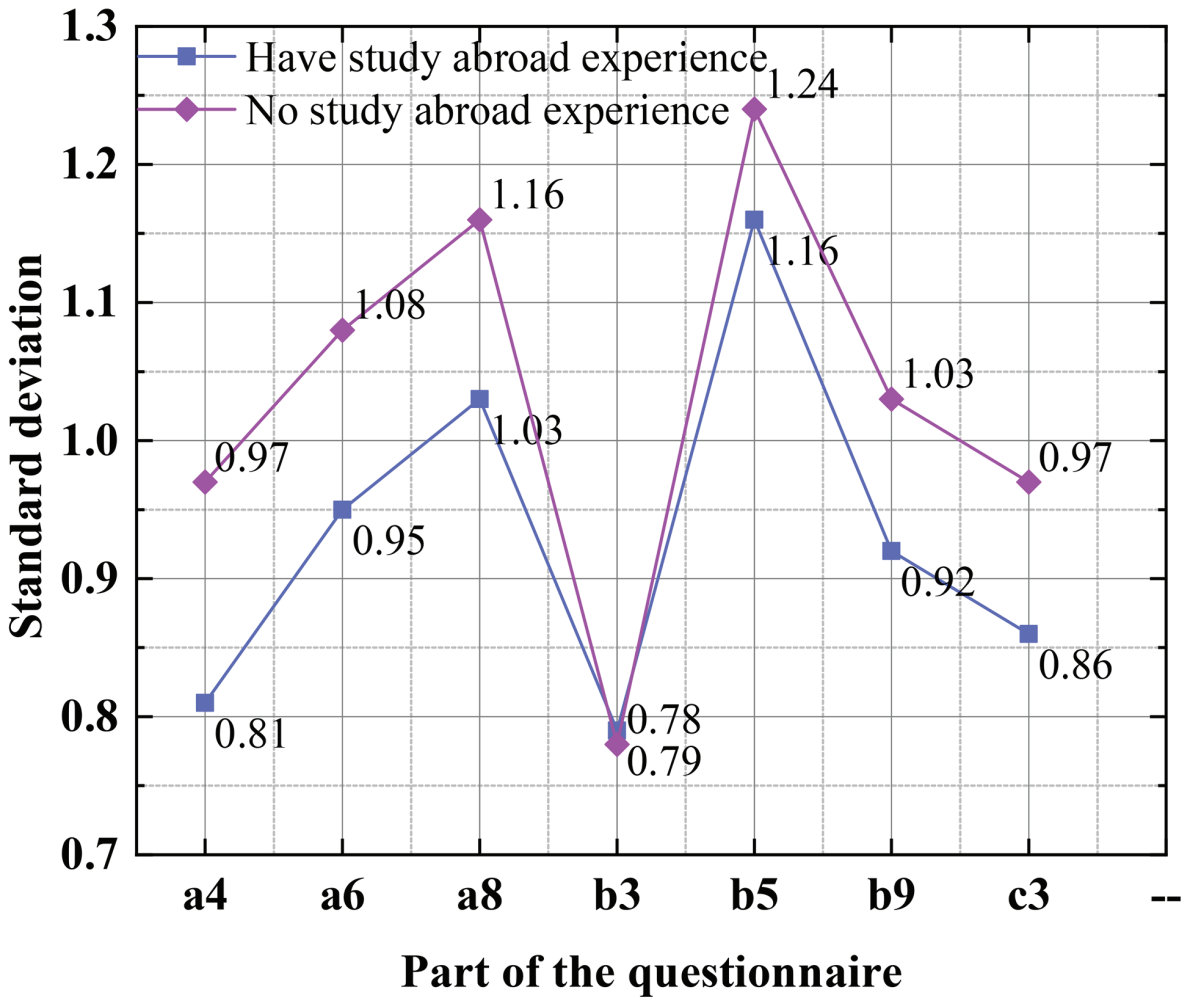
Standard deviation of the scores of the questionnaire in the questionnaire survey.

The standard deviation of the scores of the questionnaire is calculated to further discuss and analyze the score of the subjects, as shown in Figure 6.

Figure 6 shows that a more in-depth analysis of the results of the questionnaire is conducted, and the standard deviation of each question is calculated. From the curve of standard deviation, it is found that the standard deviation of the subjects with study abroad experience is smaller than those without study experience. This also verifies that the students with study abroad experience have higher scores in each dimension of the questionnaire, and they receive better entrepreneurship education, have better psychological resilience and swindler syndrome than domestic college students (Cisco, 2020).

### Reliability and Validity Analysis of the Questionnaire

**Figure 7:**
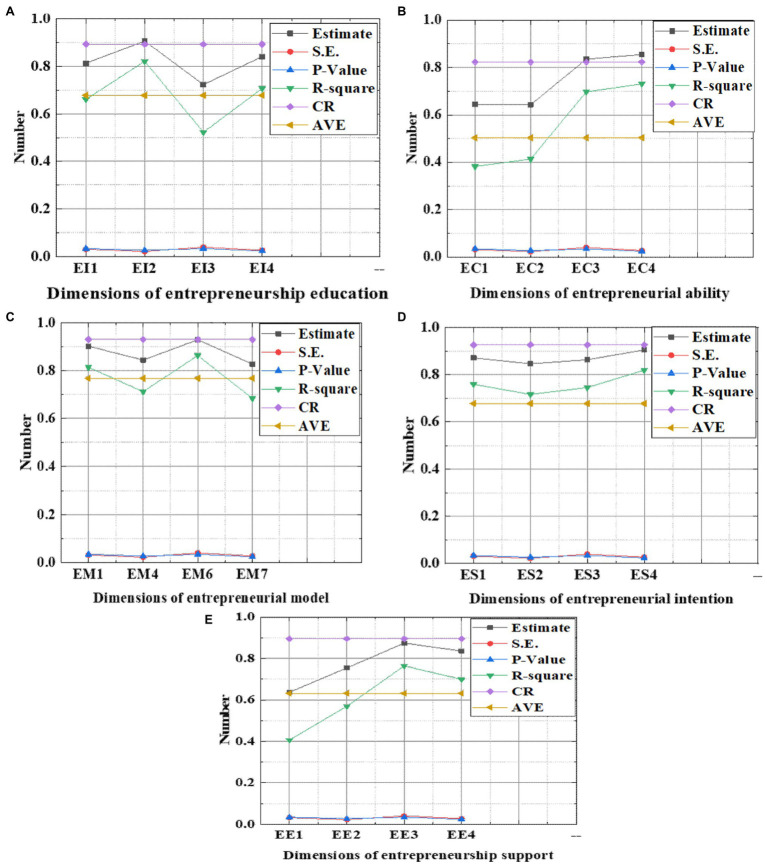
Reliability of each dimension (CR: constituent reliability; AVE: convergent validity; and S.E.: regression standard deviation). (**A**: entrepreneurship education; **B**: entrepreneurial ability; **C**: entrepreneurial model; **D**: entrepreneurial intention; and **E**: entrepreneurial support).

Figure 7 shows the reliability and validity of each dimension of the questionnaire.

Figure 7 shows the reliability of the dimension in the questionnaire. The constituent reliability is greater than 0.7, and the AVE is greater than 0.5, indicating that it has good constituent reliability and convergence validity. Combined with the calculation results in the questionnaire, it is found that the reliability values of each dimension meet the needs of analysis and research, and the questionnaire has high reliability (Dafna et al., 2020; Hofgaard et al., 2021).

### Results of Hypotheses Analysis of the Model of Entrepreneurship Education

**Figure 8:**
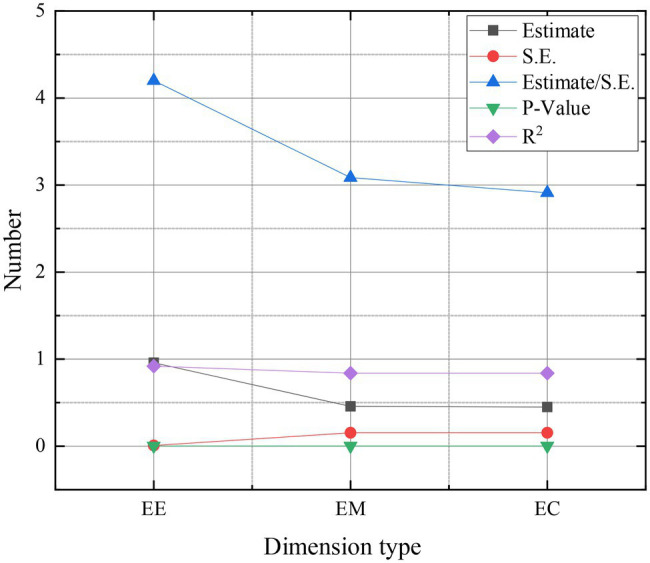
Results of the hypothesis analysis of the model (S.E.: regression standard deviation; *R*^2^: determination coefficient).

Figure 8 shows the results of the hypothesis analysis of the research model.

The fitting indexes of the mediation model are as follows: χ^2^=98.677, *df* = 62, CFI = 0.992, TLI = 0.990, and RMSEA = 0.040. In addition, the hypothesis analysis of the research model in the above figure shows that the *p* values of the mediation effect are all less than 0.05, and the mediation model is valid. The in-depth analysis of resilience is conducted from the impact of overseas Chinese returnees and entrepreneurship education. After the analysis and research, it is concluded that resilience plays a special and important role in the entrepreneurship of overseas Chinese returnees. It can make individuals have a dynamic elimination process inside so that the whole entrepreneurial process can be carried out smoothly (Qu, 2021). Through the investigation and analysis of psychological resilience, the current situation and average level of psychological resilience of overseas Chinese returnees are analyzed. And they are compared with those of the domestic students to study the relationship between psychological resilience and students’ experiences, suggestions for improving the psychological resilience of entrepreneurial students in colleges and universities are proposed. Entrepreneurship education is a process of teaching and learning. The correct concept of entrepreneurship education can make students better face the setbacks and failures in the process of entrepreneurship, stimulate entrepreneurs’ will, and enable them to safely and effectively go through the shadow of entrepreneurship (Melita et al., 2021).

Swindler syndrome is also a psychological state in which individuals do not trust their abilities when they achieve success. Through the analysis and study of the psychological resilience of these individuals, their problems are explored. Because of these problems, students’ psychologies are imperceptibly changed by carrying out the above contents, so that students can have a more accurate understanding of their abilities, face up to their own effort’s skills, and weaken their performance of swindler syndrome (Anant et al., 2018; Wang, 2021).

### Analysis of Entrepreneurship Education for Overseas Chinese Returnees Based on Psychological Resilience

**Figure 9:**
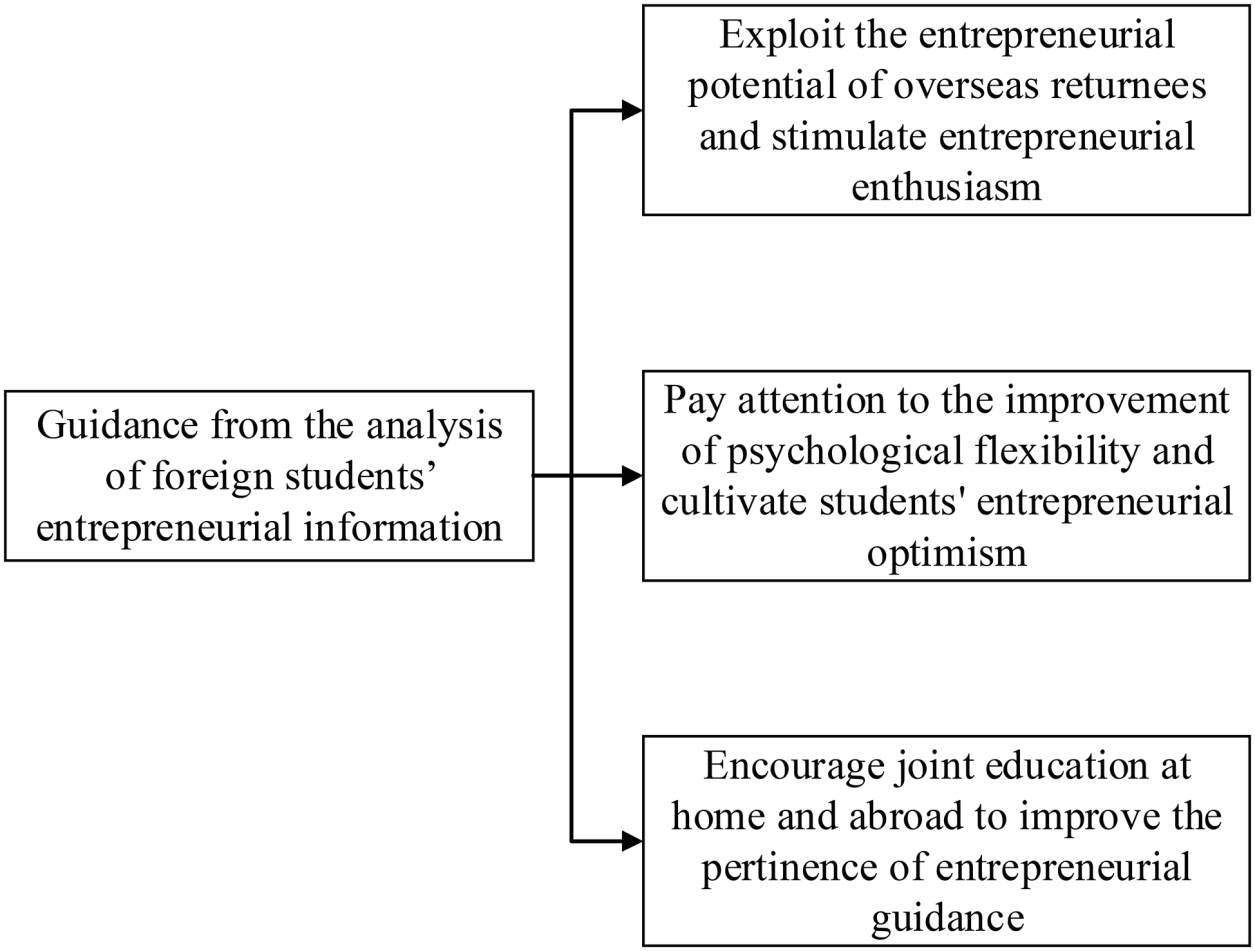
Guidance from the analysis of foreign students’ entrepreneurial information.

In summary, based on the analysis of psychological resilience on overseas Chinese returnees’ swindler syndrome and entrepreneurship education, the planning and development direction of entrepreneurship education are comprehensively summarized for college students in China. The guidance for entrepreneurship education in colleges and universities is mainly reflected in three aspects, as shown in Figure 9.

1. Policy support for entrepreneurship can not only make individuals more interested in entrepreneurial activities but also create an excellent entrepreneurial environment for entrepreneurs, enabling people with entrepreneurial ideas to gain more meaningful entrepreneurial knowledge and entrepreneurial ability. In addition, individuals who can quickly accept the integration of other cultures can better adapt to different complex environments, and get all-around entrepreneurial information. Therefore, China must strive to improve the quality of entrepreneurship education and encourage universities to open relevant courses. The support for overseas Chinese returnees’ entrepreneurship increases and relevant policies are formulated to attract overseas Chinese returnees to return to China. The examples of entrepreneurial success should be publicized to inspire more people to start businesses (Vatsayan et al., 2018).
2. The improvement of psychological resilience can make overseas Chinese returnees adapt to the new environment more quickly, have a clearer understanding and affirmation of their ability, and face cultural differences more calmly. Overseas Chinese returnees with strong psychological resilience can carry out entrepreneurial activities more successfully. Therefore, students’ psychological resilience should be strengthened to better face the frustration of entrepreneurial pressure. Entrepreneurship education in colleges and universities in China should focus on training students’ psychological resilience and promoting the development of students’ entrepreneurship education.
3. The experience of studying abroad also has an important impact on entrepreneurship. Therefore, China can carry out joint education in colleges and universities, and expand the number of exchange students. And students with entrepreneurial intentions should be encouraged to carry out entrepreneurship education from various angles (Sommarström et al., 2021).

This article uses the questionnaire survey method to conduct research. Many questions are designed in the questionnaire for the surveyed students to choose. As shown in Table 1, it shows a summary of the questions designed in the questionnaire in this article.

**Table 1:** Some questions in the questionnaire.

Question number	Question content and options (1 totally disagree; 2 disagree; 3 neutral; 4 agree; 5 totally agree)
a4	There are many related activities in schools as well as in the workshops.
a6	Teachers guide and encourage students to start a business.
a8	Schools provide supplementary entrepreneurial resources and preferential policies for students.
b3	You can be brave enough to respond to the challenges and difficulties in the process of entrepreneurship.
b5	You believe you can cope with the difficulties encountered in the entrepreneurial process and achieve the goal.
b9	Believe that there are peer partners to help you in the pressure of entrepreneurship.
c3	You usually seek prospective entrepreneurial opportunities.

As shown in Table 1, the questions in the questionnaire design of this article basically revolve around the student’s learning process and entrepreneurial process, involving the impact of school management, the teacher’s education, and the impact of their own factors, and comprehensively cover the students’ entrepreneurial process Specific influencing factors.

## Conclusion

With the acceleration of China’s economic transformation, many overseas Chinese returnees choose to return China for employment, but China’s entrepreneurial rate is still at a low level. In this case, it is necessary to improve the entrepreneurial intention of Chinese people. And overseas Chinese returnees are taken as the research subjects to explore their entrepreneurial intention. The purpose of this study is to conduct research and entrepreneurship education on overseas Chinese returnees with swindler syndrome through psychological resilience. First, a questionnaire is used to investigate the entrepreneurship education of the overseas Chinese returnees with the swindler syndrome after a lot of literature is reviewed. Through the analysis of the results of the survey data, the role of resilience in the process of overseas Chinese returnees’ entrepreneurship is summarized. The results show that the resilience and innovation ability of the overseas Chinese returnees are higher than those of students without studying abroad. And they have more advantages in entrepreneurship, and their resilience is stronger than other colleges students, and have confidence in their excellence and ability. It is concluded that resilience promotes the development of entrepreneurship education. The shortcomings of the study are that the analysis of the related factors of entrepreneurship needs to be deepened, and there are many other influencing factors of entrepreneurship that are not included in the study, such as social factors and family factors. A more perfect model of entrepreneurship education for overseas Chinese returnees will be implemented in the follow-up research. This study provides theoretical data for China’s entrepreneurship education, promotes the development of entrepreneurship education in China, and attaches great importance to psychological resilience in entrepreneurship education. Also, it provides a reference for the research on the entrepreneurship education of overseas Chinese returnees, which will greatly promote the entrepreneurial development of college students.

## Data Availability Statement

The raw data supporting the conclusions of this article will be made available by the authors, without undue reservation.

## Ethics Statement

The studies involving human participants were reviewed and approved by Washington State University Ethics Committee. The patients/participants provided their written informed consent to participate in this study. Written informed consent was obtained from the individual(s) for the publication of any potentially identifiable images or data included in this article.

## Author Contributions

All authors listed have made a substantial, direct and intellectual contribution to the work, and approved it for publication.

## Conflict of Interest

The authors declare that the research was conducted in the absence of any commercial or financial relationships that could be construed as a potential conflict of interest.

## Publisher’s Note

All claims expressed in this article are solely those of the authors and do not necessarily represent those of their affiliated organizations, or those of the publisher, the editors and the reviewers. Any product that may be evaluated in this article, or claim that may be made by its manufacturer, is not guaranteed or endorsed by the publisher.
